# Obstetrical Complications and Outcome in Two Families with Hereditary Angioedema due to Mutation in the F12 Gene

**DOI:** 10.1155/2010/957507

**Published:** 2010-05-13

**Authors:** Olivier Picone, Anne-Claire Donnadieu, François G. Brivet, Catherine Boyer-Neumann, Véronique Frémeaux-Bacchi, René Frydman

**Affiliations:** ^1^Université Paris-Sud, 92141 Clamart, France; ^2^AP-HP, Service de Gynécologie-Obstétrique et Médecine de la Reproduction, Hôpital Antoine Béclère, 92141 Clamart, France; ^3^INSERM, U 782, 92140 Clamart, France; ^4^AP-HP, Service de Réanimation Médicale, Hôpital Antoine Béclère, 92141 Clamart, France; ^5^INSERM, U 764, 92140 Clamart, France; ^6^AP-HP, Service d'Hématologie Biologique, Hôpital Antoine Béclère, 92141 Clamart, France; ^7^INSERM, U 770, 94270 Le Kremlin Bicêtre, France; ^8^AP-HP, Département d'Immunologie, Immunologie Biologique, Hôpital Européen Georges Pompidou, Université Paris V, 75908 Paris, France; ^9^INSERM, U 255, 75015 Paris, France

## Abstract

*Backgroud*. Hereditary angioedema (HAE) is characterized by recurrent swelling of the skin, the abdomen (causing severe acute pain), and the airways. A recently discovered type caused by mutations in the factor XII gene (designated as HAE type III) occurs mainly in women. Estrogens may play an important role, but few obstetrical complications have been reported. *Case*. We report the symptoms and obstetrical complications of women in two families with HAE attributable to the p. Thr328Lys mutation in the *F12* gene. Clinical manifestations included acute and severe maternal abdominal pain, with transient ascites, laryngeal edema, and fetal and neonatal deaths. Patients had normal C4 levels and a normal C1 inhibitor gene. Administration of C1-inhibitor concentration twice monthly decreased the attack rate in one mother, and its predelivery administration (1000 U) led to the delivery of healthy girls. *Conclusions*. Obstetricians and anesthesiologists should be aware of this rare cause of unexplained maternal ascites and in utero or fetal death associated with edema.

## 1. Introduction

Hereditary angioedema (HAE), a rare autosomal dominant disease, manifests as recurrent episodes of localized edema, which can involve the larynx and lead to upper airway obstruction and even fatal asphyxiation. Gut involvement results in occlusion, anorexia, vomiting, abdominal pain, and ascites. Symptoms usually begin at school age, and diagnosis is based on a familial history and laboratory data. In some cases, however, HAE occurs only during pregnancy, as severe attacks of abdominal pain [1–3]. Moreover, a normal complement C4 concentration [4] was previously considered to rule out HAE. Accordingly, patients without any positive family history or with normal C4 values [5, 6] often did not undergo assessment of C1-inhibitor protein levels and function [7]. The classic forms of HAE, types I and II, are caused by abnormal C1-inhibitor function, due to mutations in the gene encoding this inhibitor (SERPING1) [8, 9]. A third type, initially designated estrogen-dependent HAE, has recently been described in patients with normal C1-inhibitor concentration and function. This new type (called type III HAE) mostly affects women and appears to be triggered by exposure to high estrogen levels. It may also, however, occur before puberty, in postmenopausal women, and, rarely, in men [10–15]. In 2006, Dewald and Bork identified the causative mutations in the coagulation factor XII gene that lead to abnormal kinin generation [16]. In some families, all affected individuals in some families were reported to be women, but in others, men were also affected [11–13, 15]. Although high estrogen levels in these families may have influenced the frequency of attacks, exacerbations, or improvements, few obstetrical complications have been reported. No miscarriage or fetal or neonatal death was mentioned in a recent large cohort [15], although Bouillet et al. did report a case of in utero death in one French family [17]. We report here obstetrical complications observed in two families with a missense mutation in the factor XII gene responsible for recurrent attacks of severe abdominal pain, transient maternal ascites, and fetal and neonatal deaths in two carriers from one of the families.

## 2. Cases

### 2.1. Family 1

The patient, a 31-year-old Jewish woman, was followed at our tertiary center during her fourth pregnancy in a series of complicated pregnancies. During the first, ascites developed at 31 weeks of gestation (WG), and the fetus died in utero, of an unidentified cause (no chromosomal or morphological or placental anomalies). The simultaneous occurrence of these two events led us to the conclusion that they were linked. The second and third pregnancies were very painful, with abdominal attacks (pains and vomiting) at 10-day intervals. Cesarean deliveries were performed in both pregnancies, during abdominal crises with ascites, due to the risk of fetal death, and led to the birth, at 33 and 35 WG, of two healthy girls. Preeclampsia, antiphospholipid syndrome, thrombophilia, afibrinogenemia, and factor XIII deficiency were all ruled out. During the last pregnancy, the patient was admitted on several occasions for vomiting, abdominal pain, and ultrasound-documented ascites, associated once with acute kidney injury. Familial Mediterranean fever was ruled out, both by recurrence during colchicine treatment and by genetic analysis. At an extensive reinterview, the patient reported that abdominal symptoms occurred two days after swelling of the face and extremities, that these symptoms occurred only during pregnancy or when she used oral contraception, and generally it resolved within 72 hours. Testing showed that complement C4- and C1-inhibitor antigen levels were normal, whereas C1-inhibitor activity, assessed in an accredited laboratory (VFB) at 30 and 34 WG, was low (33% and 25% of normal values, resp.) (Table 1).

 As HAE was suspected, infusion of a C1-inhibitor concentrate protocol (500 U, twice monthly) was initiated. After three courses of uneventful and efficacious treatment, a severe abdominal attack with abundant ascites at 35.5 WG led to the infusion of C1-inhibitor concentrate (1000 U Berinert P; CSL Behring, Marburg, Germany) and the extraction immediately thereafter of a healthy girl (2500 g). No episodes occurred either after delivery or during the three-year followup period, and C1-inhibitor function, measured at month 15, was normal (Table 1). 

 The patient reported that her maternal grandmother and a maternal first cousin had complained of repeated episodes of facial swelling. Her mother had reported recurrent abdominal pain and vomiting without skin swelling, both exclusively during pregnancy. This family history is suggestive of HAE. Two maternal aunts were unaffected and had had normal pregnancies, but a third maternal aunt had three pregnancies (two boys and twin girls) complicated by recurrent severe vomiting without skin swelling and unexplained neonatal deaths. Subsequent testing showed her to have a normal concentration of functional C1-inhibitor. One cousin also had recurrent abdominal pains and ascites during her pregnancy. These clinical presentations lead us to the diagnosis of type III HAE.

### 2.2. Family 2

The patient, a 37-year-old Arab woman, was transferred to our center at 33 WG during her second pregnancy for suspected HAE. She had previously suffered a spontaneous first-trimester miscarriage of unknown etiology. She complained of edema of the face and larynx and abdominal pains, all occurring only during pregnancy. Her mother had died, possibly of laryngeal edema, during her fourth pregnancy. The patient's serum C4 component and C1-inhibitor antigen concentrations were normal, but her C1-inhibitor activity was 33% of the normal level (Table 1). The delivery of a healthy girl (4370 g) was induced at 41 WG for obstetric reasons after C1-inhibitor perfusion (1000 U). The peripartum period was uneventful, and functional C1-inhibitor levels reached normal values.

### 2.3. Genetic Analysis

Blood samples were taken after written informed consent. DNA was extracted and the gene encoding complement C1-inhibitor was analyzed and shown to be normal. We then searched for the recently described p. Thr328Lys mutation of F12 associated with this type of HAE. Direct sequencing of forward and reverse strands was performed with the BigDye terminator cycle sequencing kit (Applied Biosystems), using the same primers as for PCR amplification (5′-3′ F AAGCGCGGAACTGGGGAC; R CCG GCTGGCCGGAATCTA). 

**Figure 1:**
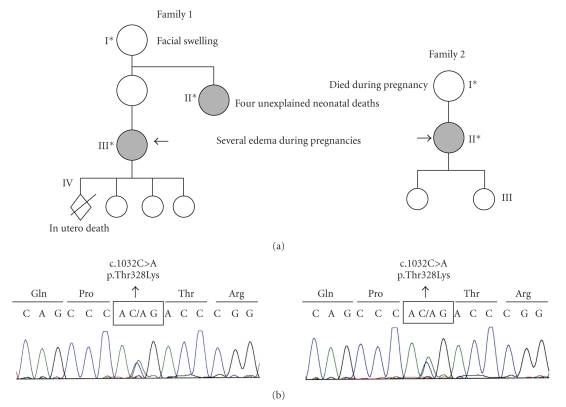
Mutation in the F12 gene in two pedigrees. The pedigree of the two families is shown. Individuals are identified by Arabic numerals within each generation (roman numerals). Affected individuals are indicated with asterisks. A heterozygous mutation was found in subjects with solid symbols. Electrophoregrams correspond to the DNA sequence surrounding the mutated nucleotide in the F12 gene. In exon 9, a C > G heterozygous missense mutation was noted in both patients. Direct sequencing of forward and reverse strands was carried out with the BigDye terminator cycle sequencing kit (Applied Biosystems), using the same primers as for PCR amplification (5′-3′ F AAGCGCGGAACTGGGGAC; R CCG GCTGGCCGGAATCTA).

 This mutation was identified in both cases and in the affected aunt (family 1). Blood samples were not available from the grandmother, mother, unaffected aunts, or cousins of the first case (Figure 1).

## 3. Discussion

This report establishes a diagnosis of atypical HAE during pregnancy in two women, based on clinical signs and a positive family history, despite late onset and normal C4 concentrations, and transient deficiencies of C1-inhibitor function. 

 Most commonly, the symptoms of HAE due to C1-inhibitor deficiency begin at school age, worsen around puberty, and are due to mutations resulting in abnormal C1-inhibitor levels or function, associated with a low serum C4 concentration [1]. A new type of HAE with normal C1-inhibitor concentration and function, occurring almost entirely mostly in women, has been recently described [10, 11]. The symptoms and course of this type of HAE, designated as type III HAE, seem to differ from the classic forms (type I and type II HAE) by its later onset, longer disease-free intervals, more frequent facial swelling and tongue involvement, and less frequent attacks of abdominal pain [12]. Obstetrical complications were not reported in the patients in these studies or in those of a large Italian pedigree [14]. In our patients, HAE was manifested mainly during pregnancy, the clinical features were transient but recurrent maternal ascites, abdominal pain, and laryngeal edema. Fetal and neonatal deaths were observed in two carriers of the F12 gene mutation of one pedigree. To date, only one other in utero death has been described in a woman with HAE caused by an F12 mutation [17]. 

 We suggest that when ascites occurs during pregnancy and is due neither to an obstetric cause such as preeclampsia or mirror syndrome nor to a standard nonobstetric cause, HAE should be suspected and the patient's family history investigated [18]. Screening to confirm suspected cases of HAE can measure the C4 concentration and C1-inhibitor antigen levels, but if clinical suspicion is strong, C1-inhibitor function must be assessed, followed by genetic tests if necessary [7]. Unfortunately these investigations, performed only in reference laboratories, are time consuming. Furthermore, although most HAE cases are caused by mutations of the C1-inhibitor gene, which leads to C1-inhibitor deficiency and low C4 levels (type I or type II HAE), HAE may also occur in patients with normal C4 levels and normal C1-inhibitor functions [5, 6, 10–16] and in women with transient C1-inhibitor deficiencies during pregnancy, as in our cases and another report [17]. When no mutation was detected in our patients for the gene encoding C1-inhibitor, we searched for a mutation in the coagulation factor XII gene. In 2006, Dewald and Bork demonstrated that mutations of this gene may be related to this novel form of HAE with normal C1-inhibitor levels and function [16]. The occurrence of this mutation raises questions about the mechanism underlying this novel type of HAE, designated as type III. Cichon et al. suggested that plasma factor XII activity might increase, while normal factor XII procoagulant levels are maintained as in our cases (results not shown), and that this could lead to increased bradykinin production and vascular permeability resulting in angioedema. Others, however, could not replicate these results during the symptom-free interval between attacks [18–21]. Furthermore, the positive regulation of factor XII gene transcription by estrogens may explain the strong influence of estrogens on the disease in some women [22]. Activation of the kallikrein-kinin system (or contact system), which induces increased bradykinin formation, appears to be the main mechanism in the pathophysiology of the trigger of HAE C1 inhibitor deficiency symptoms. If we consider that the trigger factors are similar in the three types of HAE, the contact system may be also involved in the physiopathology of HAE with F12 gene mutations [15, 23]. 

 In clinical practice, HAE diagnosis should be based on clinical symptoms and confirmed by complement assessment and genetic analyses could be limited to cohort studies or for elucidation of the pathophysiology. Treatment of HAE during pregnancy presents special problems, particularly when HAE is clinically suspected and laboratory results are confusing or not yet available. In cases of a potentially fatal attack, an infusion of C1-inhibitor concentration (1000–1500 U) should be administered immediately even if the diagnosis of HAE is only presumptive (at least in the countries where this treatment is widely available) [4, 7]. Generally, in cases of C1 inhibitor deficiency, a positive response generally occurs within two hours in 95% of attacks; if symptoms persist, an additional infusion may be administered and alternative diagnoses considered [1]. Unfortunately, the efficacy of CI inhibitor concentration for type III HAE caused by mutations in the F12 gene varies substantially from very to not at all effective [15]. If attacks occur long before delivery, prophylaxis should be discussed after any emergency care. Androgens are theoretically contraindicated and are, in any case, ineffective [23]. Tranexamic acid, not recommended, may trigger thrombotic events [4]. Kallicrein inhibitor, bradykinin B2 receptor inhibitors (Icatibant), or recombinant C1-inhibitor may prove useful in the future but are unlikely to be allowed during pregnancy soon [7, 24, 25]. Currently, for type I and type II HAE, regular C1-inhibitor replacement therapy can be offered until delivery (once or twice a week), but it is difficult to predict the requirements of individual woman [4, 7, 26, 27]. For the peripartum period of type I or type II HAE, the safest approach appears to be the systematic administration of a predelivery infusion of 500 or 1000 U of C1 inhibitor concentration [4]. Unfortunately, no recommendations yet exist for type III HAE. However, in view of Bouillet's experience and the efficiency of the administration of 1000 U of C1-inhibitor concentration in the two patients reported here, this predelivery infusion may be useful during the peripartum period [17]. Furthermore, because trigger factors appear to be the same as those of C1 inhibitor deficiency, regional analgesia is to be preferred to endotracheal intubation to avoid laryngeal edema if operative delivery is undertaken [4, 15]. 

 Obstetricians and physicians should be aware of the maternal and fetal complications of HAE, a rare disease that may occur only during pregnancy. It may be suspected on clinical grounds and may require specific emergency care or prophylaxis.

## Figures and Tables

**Table 1:** Complement profiles in patients. The diagnosis of patient 1 was reviewed two years after the fourth pregnancy. Complement was quantified for patient 1 at 30 and 34 weeks of gestation (W) and 15 months after the pregnancy (M15) and for patient 2 at 29 weeks and 33 weeks of gestation and 2 months after the pregnancy (M2). Plasma concentrations of C1 inhibitor (C1 inh), C4, and C3 were determined by nephelemetry (Dade Behring). Normal values ranged between 170 and 540 mg/L for C1-inhibitor (C1 Inh), 93 and 380 mg/L for C4, and 660 and 1250 mg/L (±2 SD) for C3. CH50 were determined according to standard procedures. Results are expressed as percent of the CH50 of the reference plasma pool (obtained from one hundred healthy blood donors). C1-inhibitor function (C1 inh fx) was assessed in a chromogenic assay (Technochrom, Biolys, Taluyers, France).

			Patient 1			Patient 2		
		Units	30 w	34 w	M15	29 w	33 w	M2
CH50	70–130	%	155	144	131	114	120	107
C3	660–1250	mg/L	1470	1200	989	1270	1310	926
C4	93–380	mg/L	425	352	320	215	257	214
C1 Inh	170–540	mg/L	202	186	239	148	161	190
C1 inh fx	70–130	%	33	25	101	33	35	84
